# False-positive HIV in a patient with SARS-CoV-2 infection; a case report

**DOI:** 10.1016/j.amsu.2021.103027

**Published:** 2021-11-06

**Authors:** Rawezh Q. Salih, Gasha A. Salih, Berwn A. Abdulla, Abdulla D. Ahmed, Hawbash R. Mohammed, Fahmi H. Kakamad, Abdulwahid M. Salih

**Affiliations:** a Smart Health Tower, Madam Mitterrand Street, Sulaimani, Kurdistan, Iraq; b Kscien Organization, Hamdi Str, Azadi Mall, Sulaimani, Kurdistan, Iraq; c Gaziosmanpasa University, Faculty of Medicine, Tokat, Turkey; d Univeristy of Human Development, College of Health Science, Sulaimani, Kurdistan, Iraq; e University of Sulaimani, College of Medicine, Madam Mitterrand Street, Sulaimani, Kurdistan, Iraq

**Keywords:** SARS-CoV-2, COVID-19, HIV, Cross-reactivity, False-positive

## Abstract

**Introduction:**

A small portion of Corona Virus disease-2019 (COVID-19) cases associated with co-infections, however occasionally they turn out to be false positive due to possible cross-reactivities. The current report aims to present a rare case of false-positive human immunodeficiency virus (HIV) in a COVID-19 patient.

**Case report:**

A 32-year-old female complaining from thyroid problems referred for thyroid operation. She had mild symptoms of COVID-19. Her preoperative laboratory findings were normal, except for HIV screening test which was repetitively positive. RNA PCR was performed to confirm the diagnosis of HIV, it revealed a negative result. The patient underwent thyroidectomy as planned and was given the required supportive treatment to recover from COVID-19. Two-month follow up revealed that she was negative for COVID-19 on PCR testing, and HIV immunoassay test was no longer positive.

**Discussion:**

Due to structural similarities between the spike protein chains of SARS-CoV-2 and some other viruses such as dengue, Zika, and other closely related coronaviruses (SARS-CoV, MERS-CoV), the protein can potentially interfere with the immunoassay tests. Although HIV immunoassay tests have high sensitivity and specificity, false-positive results have been reported, such as in the case of Epstein Barr virus, Influenza vaccination, and the Australian COVID-19 vaccination.

**Conclusion:**

Similarity between HIV and SARS-CoV-2 spike proteins can lead to antibody cross-reactivities, yielding false-positive results on immunoassay screening tests.

## Abstract (author highlights)

• There is a similarity between the spike protein of SARS-CoV-2 and some other viruses.
• The similar spike protein of HIV and SARS-CoV-2 can lead to antibody cross-reactivity.
• This report presents a rare case of false-positive HIV in a patient with SARS-CoV-2 infection.

## 1 Introduction

Severe acute respiratory syndrome coronavirus 2 (SARS-CoV-2) is a novel viral pathogen that causes the coronavirus disease-2019 (COVID-19), it first emerged from Wuhan, China in late 2019, and became a pandemic soon after [1]. The infection is highly transmissive and primarily spreads through sneezing, coughing, or talking within close proximities, hence it caused major social distancing in populated areas [2]. Even though the majority of COVID-19 cases are asymptomatic or have mild symptoms (such as, dry cough and fever), a minority of the cases (14%) can develop acute respiratory distress syndrome (ARDS), especially in the presence of the risk factors including; old age, hypertension, and diabetes [1]. It is regarded as a systemic disease and involves several organs of the body [3]. Amongst COVID-19 cases, 7.2% are reported to be associated with other bacterial, fungal, or viral pathogens, which in turn can affect survivability and the approach used to treat these cases [4,5]. However, false-positive results of co-infections and misdiagnosis in the setting of COVID-19 have been occasionally reported in the literature, such as Dengue virus, indicating potential cross reactivity between SARS-CoV-2 and some other pathogens [6]. It is important to consider false-positive results in these cases, as they might result in unsuitable management and infection control [7]. False-positive result of human immunodeficiency virus (HIV) with SARS-CoV-2 infection is highly unique with only a few reported cases being present in the literature [8].

The current paper aims to present an exceedingly rare case of false-positive HIV with confirmed SARS-CoV-2 infection. SCARE 2020 guidelines have been taken into account in the writing of this report [9].

## 2 Case presentation

**Patient information:** A 32-year-old married female working as a housewife was referred for total thyroidectomy as she had been diagnosed as a case of papillary thyroid carcinoma (PTC).

**Clinical findings:** Upon examination, the patient had an ill-defining anterior neck mass, she also had common mild symptoms of COVID-19, such as coughing, dyspnea and fever.

**Diagnostic approach:** Preoperative laboratory findings showed normal levels of blood glucose, CBC, TSH, calcium, and blood urea. Polymerase chain reaction (PCR) for the nasal swab confirmed the diagnosis of COVID-19. However, anti-HIV antibody titer using Roche Cobas E411 analyzer was positive (repeated 3 times). To confirm the diagnosis of HIV, RNA-PCR test was performed which surprisingly revealed a negative result.

**Therapeutic intervention:** With full viral precaution, under general anesthesia, the patient underwent total thyroidectomy as planned and was given the required supportive treatment to recover from COVID-19. The histopathological examination of the specimen confirmed the diagnosis of the papillary thyroid cancer.

**Follow-up and outcome:** The patient was followed up after two months, PCR testing showed that she was negative for COVID-19 and her HIV immunoassay test was no longer positive.

## 3 Discussion

COVID-19 is considered as a systemic disease as it affects different parts of the body, with a small percentage of these cases being presented with other microbial co-infections which can further complicate the condition [4]. However, some reports have indicated that these infections can sometimes be a false-positive, such as in the case of dengue and zika viruses [10]. False-positive result of HIV in the setting of COVID-19 is extremely rare with only 3 cases being reported in the literature [8,11].

SARS-CoV-2 is an enveloped RNA virus belonging to the Coronaviridae family, they possess transmembrane crown-like glycoprotein spikes (S) on their outer surfaces that have a crucial role in host cell recognition and penetration, and can trigger host humoral immune response, hence trigger the production of their antibody counterparts [12]. The spike protein has a large surface area and consists of an S1 receptor binding domain (RBD) and an S2 non-receptor binding domain (non-RBD) that promotes viral fusion [13]. This spike protein or at least only the RBD domain has been utilized in enzyme-linked immunosorbent assays for COVID-19 testing [14].

Due to structural similarities between the spike protein chains of SARS-CoV-2 and some other viruses such as dengue and Zika, the protein can mediate antibody cross-reactivity between SARS-CoV-2 and these viruses, as a result, it can potentially interfere with immunoassay tests and lead to false-positive diagnosis [10]. Hicks and colleagues also demonstrated that the spike protein of SARS-CoV-2 can lead to antibody cross-reactivities with other closely related coronaviruses in which they all possess the spike proteins on their outer surfaces, such as SARS-CoV, MERS-CoV, and some seasonal embecoviruses (HCoV-OC43 and HCoV-HKU1), with the former two having the highest level of cross-reactivity with SARS-CoV-2 [14].

Generally, patients with COVID-19 present with fever, dyspnea, and coughing [2]. However, prolonged fever and respiratory symptoms may also be indicative of HIV [11]. Hence, laboratory tests are required to confirm the diagnosis of the condition, or to determine the presence of co-infection as HIV which in the setting of COVID-19 has a prevalence of 1.22% [15]. Meanwhile, in 2020 Tan et al. reported 2 cases of false-positive HIV with SARS-CoV-2 infection, they used Abbot Architect multiple times for the diagnosis of HIV and still achieved the same result. On the other hand, immunoblot test was negative in both cases [11]. And later in 2021, Papamanoli and associates reported another false-positive case, again they used the Abbot Architect in their diagnosis [8]. Marking the current case, the 4th of this kind which was diagnosed via Roche Cobas E411 immunoassay testing platform.

This antibody cross-reactivity between the two viruses may be likely because the spike protein of HIV-1 shares astonishing similarity to that of SARS-CoV-2 [16]. To further support this argument, Yamaniha et al. have reported the exact opposite of the current case by presenting a false-positive SARS-CoV-2 antigen test in a patient with acute HIV infection [17].

Among population, the incidence of false-positive HIV result is between 0.0004% and 0.0007%, this is mostly determined by the specificity and sensitivity of the used diagnostic approach [18]. Although HIV immunoassay tests have been reported to have a high sensitivity and specificity of more than 99%, false results should still be considered in order to avoid unnecessary or unsuitable treatment as they do occasionally occur and can cause psychological distress in the patient [19]. Besides SARS-CoV-2, other agents have also been reported in the literature that have caused false-positive result in HIV screening tests, such as Epstein Barr virus, Influenza vaccination, and also the Australian COVID-19 vaccination which also led to its abandonment [18,20,21]. In order to avoid mismanagement, more specific confirmatory diagnosis of HIV is required in cases with possible SARS-CoV-2 infection, such as Western Blot, nucleic acid test, or RNA PCR testing [18].

In conclusion, HIV has a similar spike protein to that of SARS-CoV-2 which can lead to antibody cross-reactivities between the two viruses and cause false-positive results on immunoassay screening tests. Medical practitioners should be aware of the possibility of false-positive results in order to avoid mismanagement and avoid psychological distress to the patient.

## Patient consent

Written informed consent was obtained from the patient for publication of this case report and accompanying images. A copy of the written consent is available for review by the Editor-in-Chief of this journal on request.

## Source of funding

None is found.

## Provenance and peer review

Not commissioned, externally peer-reviewed.

## Annals of medicine and surgery

The following information is required for submission. Please note that failure to respond to these questions/statements will mean your submission will be returned. If you have nothing to declare in any of these categories then this should be stated.

## Please state any conflicts of interest

There is no conflict to be declared.

## Please state any sources of funding for your research

No source to be stated.

## Ethical approval

Approval is not necessary for case report in our locality.

## Consent

Consent has been taken from the patient and the family of the patient.

## Author contribution

Rawezh Q.Salih: biologist diagnosing the case, follow up the patient, and final approval of the manuscript. Gasha A.Salih, Berwn A.Abdulla, Abdulla D.Ahmed, Hawbash R.Mohammed, Fahmi H.Kakamad, Abdulwahid M.Salih: literature review, writing the manuscript, final approval of the manuscript.

## Registration of research studies

According to the previous recommendation, registration is not required for case report.

## Guarantor

Fahmi Hussein Kakamad is the Guarantor of submission.

## Declaration of competing interest

None to be declared.
